# Metabolic Mechanisms of Exercise-Induced Cardiac Remodeling

**DOI:** 10.3389/fcvm.2018.00127

**Published:** 2018-09-11

**Authors:** Kyle Fulghum, Bradford G. Hill

**Affiliations:** ^1^Department of Medicine, Envirome Institute, Institute of Molecular Cardiology, Diabetes and Obesity Center, Louisville, KY, United States; ^2^Department of Physiology, University of Louisville, Louisville, KY, United States

**Keywords:** exercise, heart, glucose, mitochondria, hypertrophy, cell signaling, metabokine, cardiomyopathy

## Abstract

Exercise has a myriad of physiological benefits that derive in part from its ability to improve cardiometabolic health. The periodic metabolic stress imposed by regular exercise appears fundamental in driving cardiovascular tissue adaptation. However, different types, intensities, or durations of exercise elicit different levels of metabolic stress and may promote distinct types of tissue remodeling. In this review, we discuss how exercise affects cardiac structure and function and how exercise-induced changes in metabolism regulate cardiac adaptation. Current evidence suggests that exercise typically elicits an adaptive, beneficial form of cardiac remodeling that involves cardiomyocyte growth and proliferation; however, chronic levels of extreme exercise may increase the risk for pathological cardiac remodeling or sudden cardiac death. An emerging theme underpinning acute as well as chronic cardiac adaptations to exercise is metabolic periodicity, which appears important for regulating mitochondrial quality and function, for stimulating metabolism-mediated exercise gene programs and hypertrophic kinase activity, and for coordinating biosynthetic pathway activity. In addition, circulating metabolites liberated during exercise trigger physiological cardiac growth. Further understanding of how exercise-mediated changes in metabolism orchestrate cell signaling and gene expression could facilitate therapeutic strategies to maximize the benefits of exercise and improve cardiac health.

## Introduction

Exercise promotes general metabolic wellness (1–3), improves mental health (4, 5), builds and preserves musculoskeletal function (6), and increases lifespan (7–10). These beneficial effects of exercise are related, in part, to enhanced function and health of cardiovascular tissues as well as to increased resistance of the heart to injury (11, 12). The magnitude of risk reduction for cardiovascular disease and survival afforded by exercise parallels that of not smoking (10, 13). Moreover, exercise is a core component of cardiac rehabilitation regimens, and, in patients with heart disease, it reduces cardiovascular morbidity and mortality (14–18). Nevertheless, the molecular mechanisms by which exercise improves cardiovascular health and prevents tissue injury remain unclear.

The recurrent deviations in whole body homeostasis caused by exercise drive adaptations in several organs, including brain, liver, adipose tissue, skeletal muscle, and, the topic of this review—the heart (6, 19). The idea that metabolic perturbations are important for attaining exercise-induced health benefits is consistent with a paradigm suggested first by Galen (*c* 129–210 CE), who recognized that not all movement is exercise and that exercise is most beneficial when vigorous, with “the criterion for vigorousness [defined by a] change in respiration…those movements which do not alter respiration are not called exercise” (20). Hence, with Galen, a definition of exercise and the overarching tenet that the salutary effects of exercise require significant deviations in metabolism first became apparent. Although several reviews cover the known mechanisms by which exercise regulates the health and adaptation of the heart and vasculature [e.g., (12, 21–25)], we highlight in this short review knowledge of how cardiac metabolism changes with exercise as well as recent findings of how exercise-induced changes in metabolism may drive cardiac remodeling. Specifically, we address the following questions:

(1) What kinds of exercise elicit changes in cardiac structure and function?
(2) How does cardiac metabolism change during exercise?
(3) How might exercise-induced changes in metabolism promote cardiac adaptation?

## What kinds of exercise elicit changes in cardiac structure and function?

Cardiac adaptations associated with exercise were first documented in 1899. Physical examination using auscultation and percussion revealed that Nordic skiers (26) and university rowers (27) had increased cardiac dimensions. The latter study highlighted that “the period of greatest enlargement corresponded to the period of the most arduous work,” (27) which provided an early indication that relatively high workloads correspond with exercise-induced cardiac growth. Later studies using electrocardiography and chest radiography identified functional and structural cardiac changes caused by exercise (28–31). Subsequent echocardiographic studies further described the degree and proportional features of the exercise-remodeled heart [reviewed in (32)]. Collectively, these studies laid the groundwork for understanding how repetitive bouts of exercise stimulate adaptive changes in the heart.

### Acute cardiac responses to exercise

**Figure 1:**
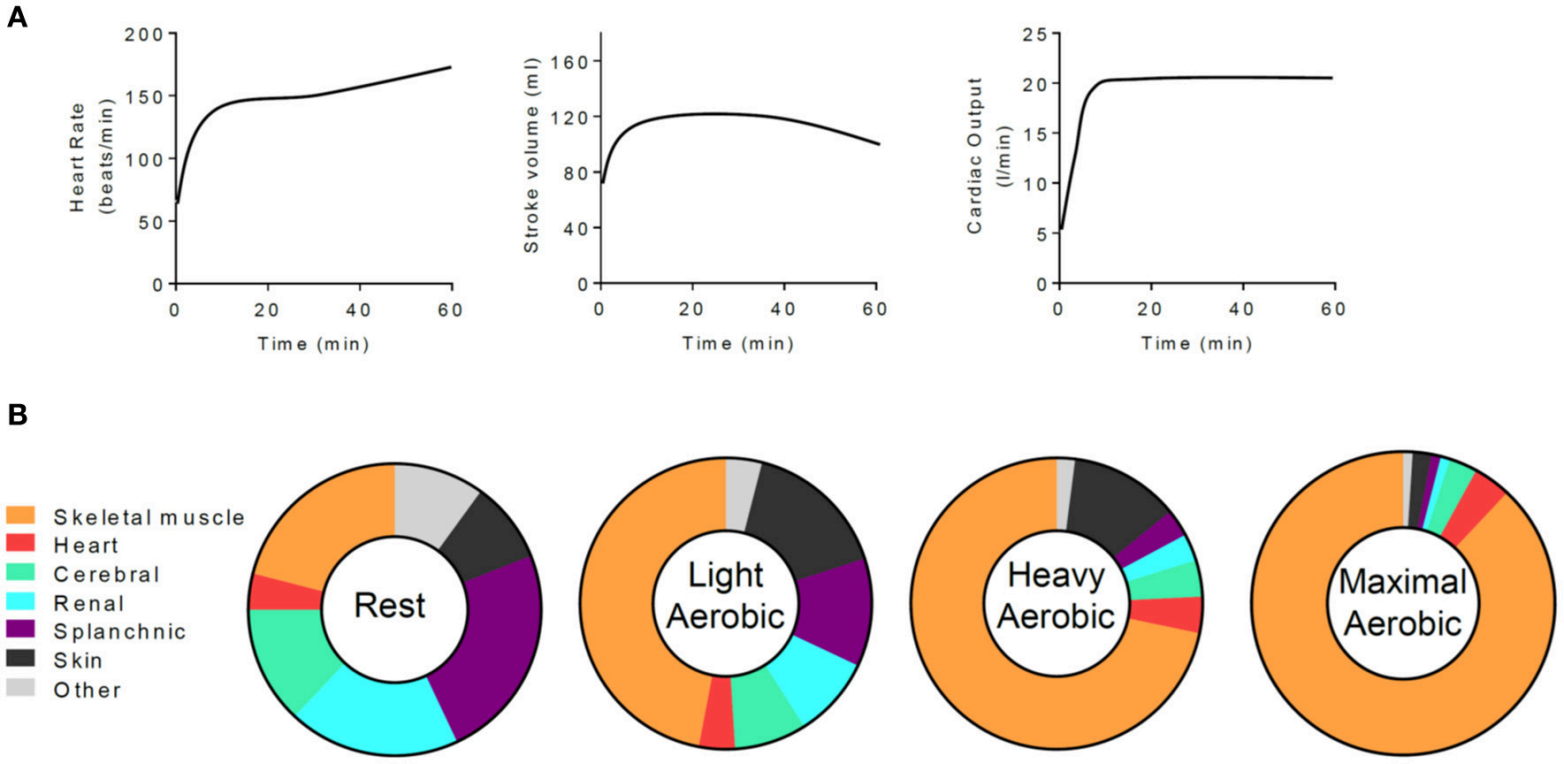
Exercise-mediated changes in cardiac function and in the tissue distribution of cardiac output. **(A)** Generalized schematic of cardiac responses to a moderate to intense, 1 h session of aerobic exercise. **(B)** Distribution of cardiac output at rest and with increasingly intense levels of exercise. Data are adapted from Plowman and Smith (38).

Increases in physical activity require changes in the distribution of oxygen and nutrients throughout the body. The increased work and ATP turnover of skeletal muscle (6) are facilitated by several integrated changes including physiological adjustments in ventilation and cardiac output as well as markedly decreased vascular resistance in skeletal muscle (19). During aerobic exercise, changes in cardiac function occur immediately and are typically associated with several phases. Heart rate and stroke volume increase upon heightened levels of physical activity, and together they augment cardiac output in a relationship defined by the Fick equation (32, 33). After a prolonged period of moderate to high intensity aerobic exercise (e.g., >20 min), cardiac output is maintained; however, heart rate tends to increase further and stroke volume begins to drop due to cardiovascular drift, a phenomenon thought to be associated with vasodilation, hyperthermia, increased blood flow to the skin, decreased filling time, and decreased plasma volume (34–37). Coordinated changes in vascular function combined with sustained augmentation of cardiac function integrate to increase blood flow to skeletal muscle, with cardiac output distribution to working muscle tracking with exercise intensity (38) (Figure 1).

Whereas the cardiac responses to endurance exercise are directly associated with the use of oxygen for ATP production in skeletal muscle, resistance exercises are more anaerobic in nature. In addition, resistance exercise generally increases blood pressure, which is due in part to mechanical restriction of blood flow during static contraction. These features of resistance exercise result in markedly different cardiac responses as compared with aerobic exercise. The modest increase in cardiac output initiated by resistance exercise is predominantly due to increases in heart rate, with virtually no change in stroke volume (39, 40). A higher number of repetitions increases heart rate and thus leads to larger increases in cardiac output (41). With heavy weightlifting, the heart must also deal with spikes in blood pressure, which can transiently reach levels of 320/250 mmHg (42) or higher. The degree to which blood pressure changes during resistance exercise appears to be a function of the degree of effort, muscle mass, and the breathing patterns commonly performed during strength training (i.e., the Valsalva maneuver) (41, 43).

### Chronic effects of exercise on the heart

**Figure 2:**
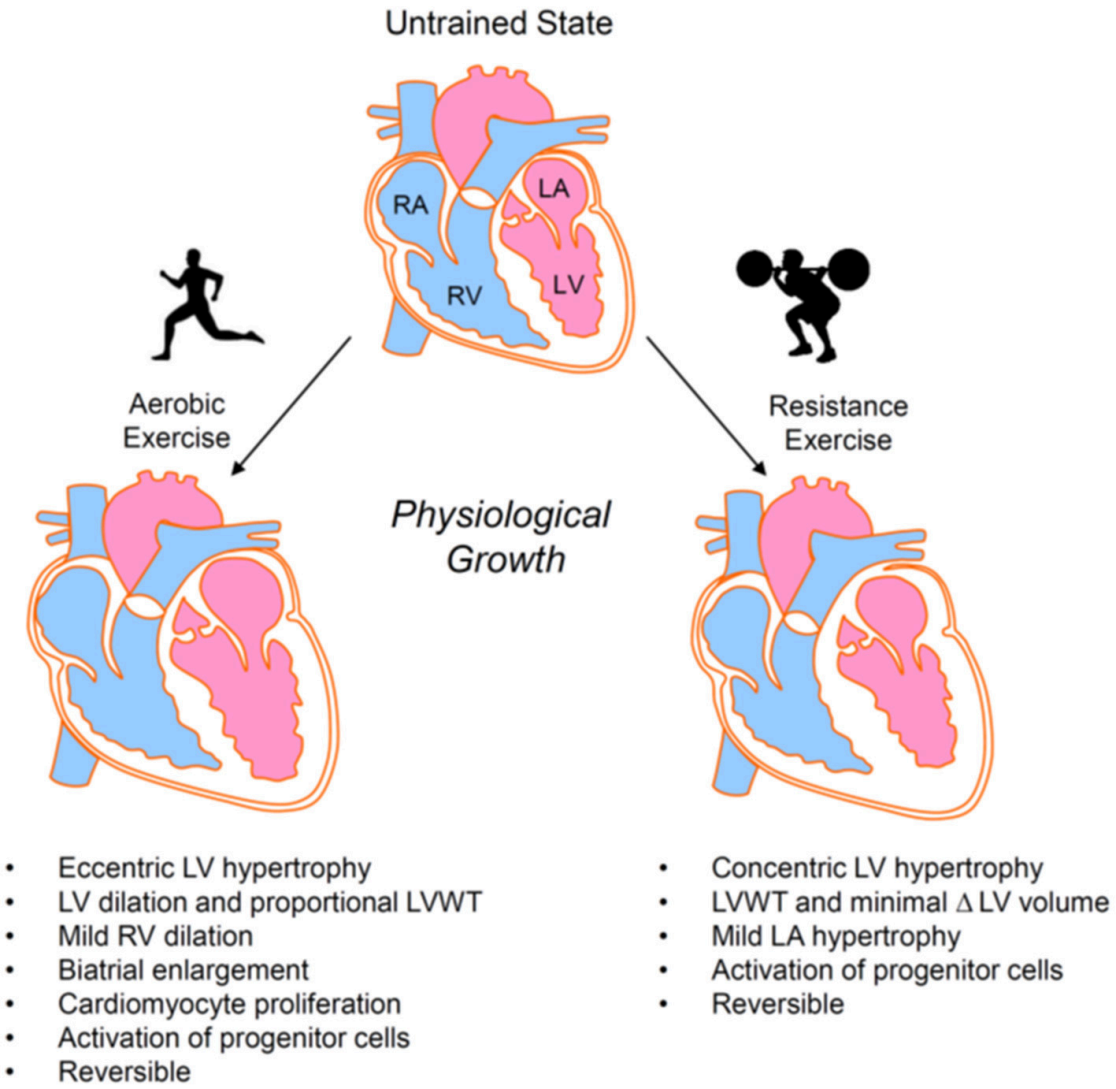
Exercise-induced cardiac growth. Aerobic and resistance exercise elicit different forms of physiological cardiac remodeling. Hypertrophic responses are primarily eccentric in nature for aerobic exercise and concentric in nature for resistance exercise. LA, left atrium; LV, left ventricle; LVWT, left ventricular wall thickness; RA, right atrium; RV, right ventricle.

Repetitive bouts of strenuous exercise can promote mild cardiac hypertrophy and/or chamber enlargement (32, 44, 45), which is typically reversible upon prolonged cessation of exercise (46–48) (Figure 2). The type and intensity of exercise determines the nature and degree of exercise-induced cardiac remodeling, with hemodynamic changes during exercise providing a stimulus for growth and chamber adaptation. Isometric or static exercises—commonly grouped as strength training (e.g., weightlifting, wrestling)—involve brief, intense periods of increased peripheral vascular resistance with little to no change in cardiac output and are associated with mild concentric hypertrophy and a normal to mildly enlarged left atrium. The increase in cardiac wall thickness appears largely caused by the parallel addition of sarcomeres within cardiomyocytes. In contrast, prolonged isotonic or dynamic aerobic exercise—generally termed endurance exercise (e.g., long distance running, cycling, rowing, or swimming)—requires sustained elevations in cardiac output and is typically associated with normal or diminished peripheral vascular resistance. Endurance exercise promotes eccentric left ventricular hypertrophy, right ventricular dilation, and biatrial enlargement [(49, 50) and reviewed in (32) and (51)]. Addition of cardiomyocyte sarcomeres in series predominates in this form of hypertrophy. Nevertheless, exercise-induced cardiac remodeling caused by endurance training has been suggested to be phasic in nature, with one study showing an initial concentric LV hypertrophy giving way to later eccentric LV hypertrophy (52) and another suggesting early increases in chamber size followed by later increases in wall thickness (53).

Although regular, intensive endurance exercise can decrease resting and submaximal heart rates [e.g., (44, 54)], the effects of exercise on other indices of cardiac function are less conspicuous. A meta-analysis of athletes participating in endurance, strength, or combined dynamic and static sports showed no major changes in systolic or diastolic function between sport type or when compared with control subjects (55). However, several studies have identified changes in diastolic function in exercise-adapted subjects. For example, endurance exercise appears to enhance diastolic function modestly (54, 56–61). In contrast, strength training could actually diminish diastolic function, as evinced by studies showing impairment of LV relaxation in American football players (61). In general, in the rested state, individuals that engage in regular exercise do not show remarkably different ejection fractions or fractional shortening values when measured by conventional echocardiography under resting conditions (54, 62–65); however, more subtle changes captured by tissue Doppler and speckle-tracking echocardiography suggest modestly enhanced systolic function in exercise-adapted subjects (66–68).

Cardiac remodeling in response to exercise appears also to involve processes beyond cardiomyocyte hypertrophy. For example, exercise increases levels of circulating progenitor cells (69–75) and cardiac-resident stem/progenitor cells (76–79), which have been implicated in augmentation of vascular density and cardiac repair (80–82). It appears that both resistance and endurance exercises activate progenitor cells [e.g., (83, 84)] and that exercise duration and/or intensity are important in the amplitude and kinetics of their activation (85–88). While the extent to which progenitor/stem cell subtypes regulate physiological cardiac growth remains unclear, their exercise-mediated activation is consistent with the angiogenesis and coronary vascular remodeling (25, 89, 90) and the improved responses to injury (91, 92) associated with exercise-induced cardiac remodeling. In addition, exercise promotes modest cardiomyocyte proliferation (78, 93), which may be important for physiological cardiac adaptation as well as for understanding the mechanisms that trigger cardiomyogenesis in the adult, mammalian heart.

### Potential deleterious effects of exercise on the heart

Although too little exercise is currently a much more serious health problem than too much exercise (94), the popularity of intense exercise (e.g., ultramarathon, CrossFit) has increased remarkably over the past 30 years (95–98). High levels of exercise can transiently increase the risk of acute cardiovascular events such as sudden cardiac death, and it can acutely diminish cardiac function, cause atrial fibrillation, trigger arrhythmias, and lead to pathological remodeling of the heart and vasculature [reviewed in (95)]. Exercise may also markedly change right ventricular morphology and function, contributing to arrhythmogenesis (99). Although young individuals that die during exercise commonly bear inherited or conditional abnormalities such as hypertrophic cardiomyopathy (100), older individuals more commonly die during exercise as a consequence of acute coronary thrombosis and myocardial infarction (101). Nevertheless, sudden cardiac death during exercise is relatively rare and has been estimated to occur in 1 per 15,000–18,000 formerly asymptomatic adults per year (102, 103).

Prolonged endurance exercise can promote “cardiac fatigue,” characterized by decreased cardiac output and ejection fraction (104, 105), although changes in cardiac function typically recover within 2 days after exercise (106). Acute decreases in cardiac function could be due to multiple factors including decreased sensitivity to catecholamines, blood volume redistribution leading to decreased venous return, and cardiomyocyte damage (95). With respect to the last possibility, mild cardiac injury during intense exercise (e.g., marathons, triathlons) is suggested by elevated levels of circulating cardiac troponins [reviewed in (95)], which are typically used to diagnose acute myocardial infarction (107), and exercise intensity is a strong predictor of elevated circulating cardiac troponin levels (108). Other biochemical indicators of cardiac dysfunction, such as B-type natriuretic peptide (BNP) and its cleaved N-terminal fragment (NT-proBNP) may be elevated up to 10-fold after endurance exercise events, but typically return to baseline levels within a few days [reviewed in (95)]. It is hypothesized that exercise-induced BNP/NT-proBNP is indicative of mild myocardial injury (109) or may be a physiological phenomenon important for cardiac adaptation (110). Endurance exercise may also promote myocardial fibrosis and increase coronary artery calcification (95), although the clinical significance of these effects in athletes remains unclear.

## How does cardiac metabolism change during exercise?

**Figure 3:**
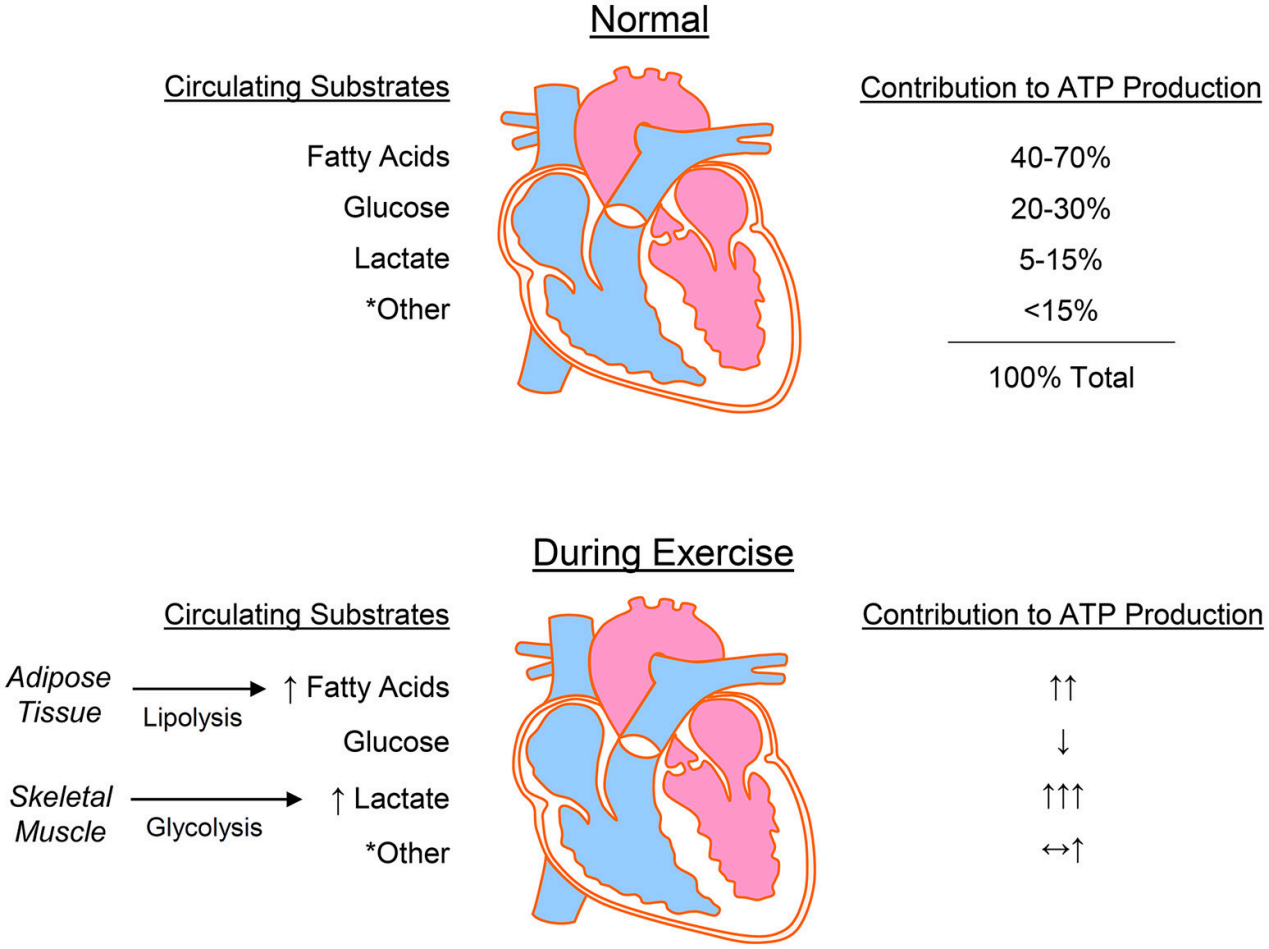
Cardiac metabolism at rest and during exercise. The heart uses numerous substrates for energy provision, with the predominant sources for ATP production being fatty acids, glucose, and lactate. During exercise, lipolysis in adipose tissue and glycolysis in working skeletal muscle increase the circulating levels of fatty acids and lactate, respectively, which are used by the heart to fuel increased energy demands. ^*^Other = ketone bodies, pyruvate, acetate, and branched chain amino acids.

The heart has a high energy demand, which requires continuous ATP generation to sustain contractile function, ion homeostasis, anabolic processes, and signaling (111–114). In normoxia, the heart fuels ATP turnover by generating >95% of its ATP from mitochondrial oxidative phosphorylation, with the remaining 5% derived from substrate level phosphorylation in glycolysis (113, 115). Although the majority of generated ATP supports contractile function, relatively large quantities of ATP are also necessary to maintain ionic homeostasis through ion pumps (116, 117). Below we review some fundamental aspects of cardiac metabolism, followed by the acute metabolic changes in the heart caused by exercise (Figure 3).

### Some fundamental aspects of cardiac metabolism

Oxidation of fatty acids is the primary contributor to ATP production in the heart, with catabolism of lactate, glucose, ketones, and amino acids fulfilling the remaining energy demand (118–120). This ability of the heart to use a myriad of substrates has led to classification of the heart as a metabolic “omnivore” capable of modulating substrate utilization in a manner dependent on numerous factors, including substrate availability, hormonal stimuli, and myocardial demand. Isotopic labeling studies in humans indicate that 84% of the FFAs entering myocytes are oxidized, with ~16% entering the triacylglycerol (TAG) pool (121). This TAG pool may contribute to ~10% of cardiac ATP production (122, 123) and also plays a central role in signaling and gene expression (124). Although fat oxidation supplies 40–70% cardiac ATP (125–131), it is also less efficient, which is due in part to fatty acid-induced uncoupling of oxidative phosphorylation (123, 132). The relative ATP yield of fats appears dependent on chain length, with long chain fatty acids yielding ~4 mol ATP/mol acetyl CoA and the shortest chain fatty acid, i.e., acetate, costing 2 mol of ATP/mol of acetyl CoA (133). Acetate is usually low in circulation (i.e., below 0.2 mM) and is unlikely to contribute meaningfully to metabolism in the normal heart; however, high alcohol consumption can increase circulating acetate levels to low millimolar concentrations (134, 135) and may under extreme circumstances contribute to cardiac energy deficits (136–139).

Substrates such as glucose, lactate, and pyruvate are generally more efficient energy sources for the heart. In the normal mammalian heart, glucose metabolism via glycolysis supplies approximately 2–8% and glucose oxidation contributes up to 30% to the ATP yield (125). Interestingly, carbon deriving from nearly half of the glucose extracted by the heart is allocated to ancillary pathways of glucose metabolism, which are important for energy storage (glycogen) or biosynthesis of cellular building blocks (e.g., nucleotides, phospholipids, amino acids) (140–145). Lactate is also a major fuel source for the heart, contributing up to 15% of ATP production (125). Lactate tracer studies indicate that the heart is a net lactate consumer (140, 146–148) and that only ~13% of glucose extracted by the heart is converted to lactate (140). Moreover, arterial lactate concentration correlates positively with myocardial lactate uptake and oxidation (141, 149, 150). In humans, lactate is a significant contributor to cardiac ATP production (141, 146), and, in dogs, it can account for up to 87% of cardiac substrate oxidation (151). In rat heart, high lactate levels contribute to nearly 40% of ATP production (150). The myocardium can also use pyruvate readily when extracellular levels are in the millimolar range; however, circulating concentrations of pyruvate are typically less than 150 μM (118), which make it an unlikely source of myocardial energy *in vivo*.

Ketone bodies such as acetoacetate and β-hydroxybutyrate have received recent attention due to their potential importance in heart failure (152–154); however, should circulating ketone bodies become highly abundant, the normal heart would be expected to increase ketone body oxidation as well. Early studies showed that high concentrations of ketone bodies (e.g., 1–10 mM) can account for nearly 80% of cardiac oxygen consumption (155), and that ketone body provision has a pronounced inhibitory effect on glucose (143, 156, 157) and fat catabolism (158, 159). Interestingly, when provided alone, ketone bodies appear to cause contractile failure (160–162); however, their availability in the presence of other substrates such as glucose may increase efficiency of the working heart (163). Such findings have advanced the idea that ketone bodies are a “superfuel” that enable efficient ATP production (164, 165). Although it has been suggested that 5–15% of ATP production in normal heart is via ketone body oxidation (125), this would depend on the levels of circulating ketones, which in the healthy, fed state are typically less than 500 μM. Although it remains to be clarified whether constitutively high levels of ketone bodies or their oxidation are healthy for the heart (166), ketone diets and ketone body supplements have been suggested to improve exercise performance and augment cardiac energy provision (167, 168).

Amino acids have a relatively minor role in ATP production in the heart; however, they are essential for processes such as protein synthesis and cell signaling. In particular, branched chain amino acids (BCAAs; comprising leucine, isoleucine, and valine) are major amino acids taken up by the heart, with uptake dependent primarily on circulating BCAA concentration (169). Because they are essential amino acids, their intracellular levels are largely dependent on import, with the L-type amino acid transporters and bidirectional amino acid transporters likely contributing to their abundance in the heart (170–172). BCAA catabolism contributes to less than 5% of myocardial oxygen consumption (173), in part because the heart expresses relatively low levels the branched chain aminotransferase enzyme and the branched chain α-keto acid dehydrogenase complex (174, 175). Nevertheless, BCAAs are important regulators of mTOR, which coordinates anabolism and processes such as proliferation, survival, and autophagy (176). Indeed, high intramyocardial levels of BCAAs are associated with cardiac hypertrophy and heart failure (177–179), and recent findings indicate that intracellular accumulation of BCAAs, via a glucose-KLF15-BCAA degradation axis, is required for mTOR activation and cardiomyocyte hypertrophy (180). High intracellular levels of BCAAs may negatively influence cardiac health by inhibiting mitochondrial metabolism (179, 181–183).

Glutamine, a “conditionally essential” amino acid, also appears to regulate the metabolism and health of the heart. In particular, it can activate mTOR in cardiomyocytes (184), and it can protect the heart from injury (185–188). Although many proliferating cells use glutamine as an oxidative fuel (189–191), the normal heart appears to produce glutamine by amidation of glutamate rather than oxidize it for energy provision (169, 192). Nevertheless, glutamine can augment myocardial oleate oxidation and triglyceride formation (193) as well as activate the hexosamine biosynthetic pathway (HBP) (194, 195).

### Cardiac intermediary metabolism in exercise

An acute increase in workload during exercise has robust effects on the metabolism of striated muscle (196). In the heart, exercise increases contractile power and oxygen consumption up to 10-fold above resting rates (24, 123). Changes in substrate utilization and ATP production during exercise are a product of the integrated effects of physiologic cues that occur with changes in circulating hormones, metabolic substrates, and hemodynamics.

An increase in myocardial workload is accompanied by increases in the catabolism of multiple substrates, in particular, fatty acids and lactate (141, 149, 197–200). During exercise, hormone-activated lipolysis in adipose tissue increases circulating FFA to levels up to 2.4 mM (201), which enhances FFA uptake and utilization (121, 147, 202). However, heightened levels of circulating FFAs are only partially responsible for increasing fatty acid oxidation because higher cardiac workloads appear sufficient to increase fat oxidation in the heart (203). Cardiac TAG utilization rates also increase considerably with exercise (198) and appear to be further stimulated by lactate availability, suggesting that lactate may stimulate TAG turnover (204). Furthermore, after exercise adaptation, genes responsible for fatty acid transport and catabolism are elevated, which may help optimize fat utilization in the heart (205–207).

Similar to free fatty acids, plasma lactate levels increase during exercise. The increase in lactate is dependent on the type of exercise, with intense exercise (e.g., 60–80% of VO_2_max) resulting in large increases in arterial lactate levels (208). During intense exercise, circulating lactate levels can increase 5–10-fold (to nearly 10 mM), which is primarily due to lactate extrusion by skeletal muscle. Under these conditions, the contribution of lactate to total oxidative metabolism may account for 60–90% of substrate utilization (149, 151, 209, 210). Although low to moderate intensity exercise (e.g., 40% of VO_2_ max) does not increase circulating lactate levels remarkably (141), the contribution of lactate oxidation to overall myocardial oxidative metabolism is higher than that compared with the sedentary state (141). Lactate may also enhance fat oxidation in the heart (199), which would increase the capacity of the heart to generate ATP under high workloads.

Although circulating levels of glucose are fairly stable compared with levels of lactate and FFAs, weightlifting and prolonged endurance exercise can decrease arterial glucose concentrations (201, 211), whereas high intensity aerobic exercise may increase blood glucose levels (197). Hemodynamic changes and increases in local and circulating catecholamines can increase the oxidation of stored glucose (glycogen) (212). Although moderate intensity exercise and increases in cardiac workload have been associated with elevations in myocardial glucose uptake and oxidation (141, 197, 199, 200), elevations in circulating concentrations of competing substrates such as lactate and FFAs may decrease glucose catabolism (197–199, 213). Moreover, studies in both humans and animal models suggest that exercise can lower oxygen extraction ratios for glucose and decrease glucose uptake and utilization (198, 213). Recent findings suggest that relatively prolonged, intense endurance exercise can decrease glucose catabolism in the heart by diminishing the activity of phosphofructokinase (214, 215). Collectively, these findings suggest that exercise can acutely increase or decrease both circulating glucose levels and myocardial use in a manner dependent on the type, intensity, or duration of exercise.

Regular exercise also promotes adaptive metabolic remodeling in the heart. Perfused mouse heart studies suggest that adaptation to exercise increases the rates of basal glycolysis (214), glucose oxidation, and fat oxidation (216); however, compared with hearts from sedentary controls, basal cardiac glycolysis has been suggested to be diminished in exercise-adapted rats, despite increases in myocardial glucose and palmitate oxidation (91). That exercise-induced changes in cardiac metabolic remodeling are dependent on exercise intensity is suggested by studies in mice in which moderate-intensity treadmill regimen showed no effect on basal glucose oxidation, palmitate oxidation, or myocardial oxygen consumption, whereas a high-intensity, interval style regimen increased glucose oxidation, diminished palmitate oxidation, and led to a net decrease in resting myocardial oxygen consumption (217). The reasons for discrepancies between studies could be due to model-specific factors (e.g., rodent strain, type of exercise) or differences in cardiac perfusion protocols (e.g., substrate levels, addition of hormones). Circadian rhythm may also account for disparate findings because it influences cardiac metabolism (218), stress responses and protein turnover (219), and inflammatory processes (220). Chronobiology remains an important consideration for understanding how exercise influences cardiac biochemistry and physiology (221, 222).

## How do exercise-induced changes in metabolism promote cardiac adaptation?

**Figure 4:**
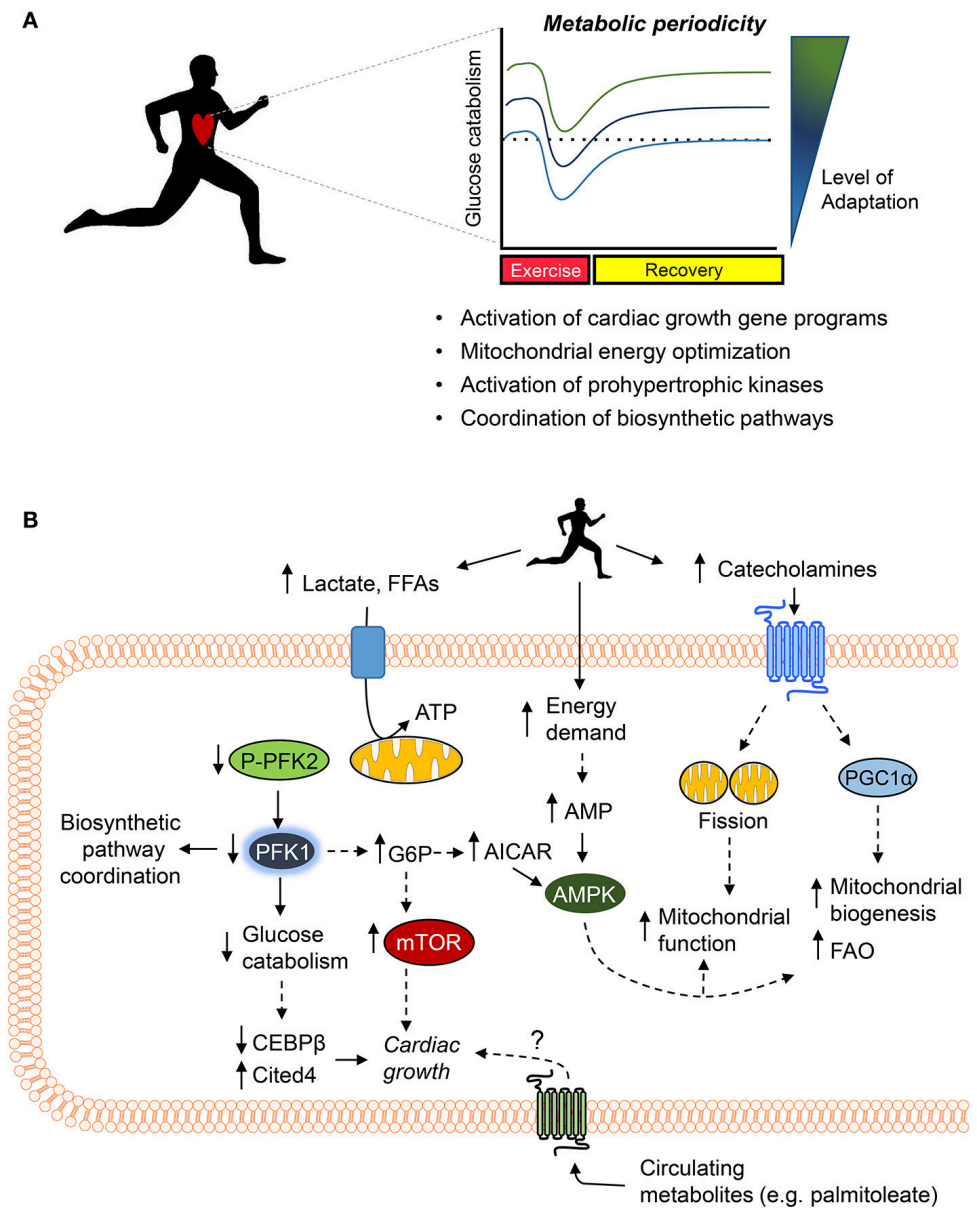
Working model of the metabolic mechanisms of exercise-induced cardiac growth. **(A)** Periodic changes in glucose metabolism and mitochondrial activity (i.e., metabolic periodicity) occurring with regular exercise promote activation of gene programs responsible for cardiac growth, regulate mitochondrial quality control and function, activate prohypertrophic kinases, and coordinate biosynthetic pathways, all of which integrate to promote cardiac growth. **(B)** Exercise increases levels of circulating cardiac substrates and catecholamines, which orchestrate changes in cardiomyocyte metabolism. Decreases in the phosphorylation of phosphofructokinase 2 (PFK2) lower phosphofructokinase 1 (PFK1) activity, which decreases glucose catabolism, coordinates ancillary biosynthetic pathways, and increases the levels of upstream glycolytic intermediates (e.g., glucose 6-phosphate, G6P) as well as increases products in the pentose phosphate pathway (e.g., AICAR). Decreases in PFK activity and glucose catabolism appear sufficient to decrease expression of *Cebpb* and upregulate *Cited4*, which promote cardiac growth. In addition, elevated levels of G6P, AMP, and AICAR could activate the prohypertrophic signaling kinase mTOR and AMPK. Catecholamine-triggered signaling cascades promote mitochondrial fission and upregulate PGC1α, which acutely increase mitochondrial function and chronically elevate mitochondrial abundance and fatty acid oxidation (FAO) capacity. Last, circulating metabolites (e.g., palmitoleate) may also contribute to exercise-induced physiological cardiac growth.

Understanding how changes in metabolism regulate cardiac adaptation to exercise presents a challenge. Metabolic pathways coordinate not only ATP production and biosynthesis, but modulate cell signaling and redox state as well (118). Nevertheless, it is clear that repetitive bouts of exercise elicit changes in metabolism that are important for coordinating gene expression in other tissues such as skeletal muscle (6). The mechanisms by which exercise-induced metabolic changes may promote cardiac adaptation are reviewed below and are summarized in Figure 4.

### Importance of metabolic periodicity in cardiac adaptation

Although episodic changes in metabolism that occur with exercise are known to play an important role in skeletal muscle adaptation (6), relatively less is known about how exercise-induced metabolic periodicity affects adaptive responses in the heart. Nevertheless, it is clear that periodic bouts of exercise stimulate metabolic processes in both cardiac mitochondria and the cytosol. For example, in mice, exercise acutely promotes fission of cardiac mitochondria, which enhances mitochondrial function; these mitochondrial changes were shown to occur in a manner dependent on adrenergic signaling (223). A relatively intense bout of exercise also decreases the activity of phosphofructokinase in mouse heart (214); however, upon adaptation to the exercise regimen and in the rested state (i.e., 24 h after the last exercise bout), apparent myocardial phosphofructokinase activity and glycolytic rate were found to be higher compared with sedentary controls (214). The acute, exercise-induced decreases in myocardial glycolytic rate appear important for cardiac growth because low phosphofructokinase activity brought forth by expression of a cardiac-specific, kinase-deficient 6-phosphofructokinase/fructose-2,6-bisphosphatase transgene in mice (Glyco^Lo^ mice) appears sufficient to partially phenocopy the exercise-adapted heart and regulate genes [e.g., *Cebpb, Cited4* (224, 225)] required for exercise-induced cardiac growth (214). Moreover, activation of the exercise gene program in Glyco^Lo^ mice occurred in the absence of Akt activation, which is thought to be required for regulating physiologic cardiac growth (21, 22, 45). These findings suggest that exercise-induced decreases in glycolysis are a proximal regulator of the cardiac growth program. Collectively, these findings indicate that exercise induces metabolic periodicity in the mitochondrial and cytosolic compartments, which regulate exercise capacity and myocardial growth.

It is likely that periodicity in mitochondrial fission and in intermediary metabolism are interconnected phenomena. In other cell systems, mitochondrial fission is important for regulating glucose and lipid metabolism (226, 227). Moreover, mitochondrial fission is important for regulating mitochondrial quality control by facilitating distribution of mitochondrial components to daughter organelles and by culling defective mitochondria via autophagy (228–230), which is increased the heart during and early after a bout of exercise (231, 232). Exercise-induced periodicity in glucose metabolism appears important for maintaining mitochondrial health because loss of periodicity, either by constitutively increasing or decreasing glucose catabolism, leads to mitochondrial dysfunction (214). Nevertheless, some mechanisms underlying mitochondrial adaptations to exercise appear to diverge from those required for cardiac growth (216, 233), which suggest the presence of distinct circuits by which metabolic changes activate the exercise gene program versus how they modulate mitochondrial health.

### Metabolic changes as a material cause of adaptation

Insight gleaned from bacteria suggest that cells coordinate growth and function via interconversion of glycolytic metabolites to biomass (234), which highlights the obvious role of metabolism as a material cause for structural maintenance and modification. It is likely that changes in ancillary biosynthetic pathway activity are also important for coupling the synthesis of structural materials to activation of the cardiac gene programs responsible for exercise-induced cardiac adaptation.

Rate-limiting steps of glycolysis, e.g., the hexokinase, phosphofructokinase and pyruvate kinase steps, are likely important for modulating biosynthetic pathways in the heart (118). These enzymes are regulated at multiple levels, with allosterism being important for acute changes in activity (235). In several cell types, the phosphofructokinase step of glycolysis regulates the pentose phosphate pathway (PPP), which is important for nucleotide synthesis and redox regulation (236–239). Modeling studies in the adult heart demonstrate that phosphofructokinase activity is particularly important for modulating the activities of the PPP and the polyol pathway (240). In cardiac myocytes, phosphofructokinase activity modulates several ancillary biosynthetic pathways, such as the PPP, the HBP, and the glycerophospholipid synthesis pathway (GLP) by directly modulating glucose carbon entry into the pathways and by indirectly regulating mitochondria-derived molecules important for building block synthesis (e.g., aspartate) (145). Furthermore, metabolomic studies indicate that phosphofructokinase activity also regulates the abundance of several amino acid and lipid metabolites in the heart (214). Much less is known about how exercise affects the hexokinase and pyruvate kinase steps of glycolysis; however, pyruvate kinase activity has been shown to be elevated in the exercise-adapted rat (241) and dog (242) heart.

There is relatively little direct knowledge of how other biosynthetic pathways change with exercise. Transient changes in readouts of HBP activity, i.e., UDP-N-acetylhexosamines or O-GlcNAcylated proteins, occur with exercise (243–246). Changes in the HBP appear important because they may regulate the function and survival of cardiomyocytes (247, 248) as well as reparative cardiac cells (249). To our knowledge, nothing is known regarding how the PPP, GLP, and SBP are influenced in the heart by exercise. While the PPP and the GLP would be thought important for regulating redox state, nucleotide biosynthesis, and phospholipid biosynthesis, the SBP modulates the levels of methyl donors required for DNA methylation reactions and could represent a critical link between metabolism, epigenetic programming, and changes in cardiac structure and function (250).

### Signaling pathways influencing cardiac adaptation

Several signaling pathways integrate to modulate cardiac metabolism and adaptive responses to exercise. Exercise-mediated increases in catecholamines promote upregulation of peroxisome proliferator-activated receptor γ coactivator 1 α (PGC1α) via β-adrenergic signaling and activation of endothelial nitric oxide synthase [reviewed in (21)]. The actions of PGC1α may be mediated via activation of nuclear receptors such as peroxisome proliferator activated receptor α (PPARα) and estrogen-related receptor (ERR) as well as nuclear receptor factor 1 (NRF1), which are known to integrate to increase fatty acid oxidation and to promote mitochondrial biogenesis. Moreover, the metabolic, structural, and functional changes occurring in the exercise-adapted heart are influenced by receptor signaling triggered by insulin-like growth factor-1 (IGF-1) (251, 252) and neuregulin-1 (78, 253), which activate the phosphoinositide 3-kinase (PI3K)/Akt pathway to promote physiologic cardiac growth (254–257) or activate a cardiomyocyte proliferative response (78, 258–260). Interestingly, cardiac glucose metabolism is influenced by catecholamines (130, 261, 262), IGF-1 (263–265), and Nrg-1 (265), which suggests that these hormones may provide additional regulation to acute or chronic metabolic changes induced by exercise.

### Metabolite signaling

Metabolite signaling is another mechanism that connects exercise-induced changes in metabolism to cardiac adaptation. In particular, glucose-derived metabolites regulate the activities of the prohypertrophic kinases mammalian target of rapamycin (mTOR) and AMP-activated kinase (AMPK) (45). The intracellular levels of glucose 6-phosphate (G6P) regulate mTOR activity in the heart (266–268), and 5-amino-4-imidazolecarboxamide ribonucleotide (AICAR), which is an intermediate of the PPP (269), stimulates AMPK (270). It is anticipated that G6P, AMP, and AICAR increase in the heart with exercise. Predictions from crossover theorem (271–274) and modeling studies (240) suggest that acute decreases in phosphofructokinase activity, such as occurs during exercise (118), would increase G6P as well as augment PPP activity, which could increase AICAR levels. In addition, the large increase in myocardial ATP demand would be thought to increase intracellular AMP levels.

Circulating metabolites are also important regulators of exercise-induced cardiac growth. Hormone-mediated adipose tissue lipolysis during exercise liberates palmitoleate (C16:1n7), which promotes cardiac growth potentially by activating G-protein-coupled receptors (GPCRs), Akt, or nuclear receptors (275). The cardiac growth-stimulating effect of palmitoleate is similar to the fatty acid-induced cardiac hypertrophy that occurs in the python heart after a large meal (276). Interestingly, FFAs not only increase acutely with exercise (201), but they appear to remain elevated in the exercise-adapted state as well (277, 278); hence, they could stimulate the signaling required to sustain cardiac adaptations. Given that numerous metabolites have cognate GPCRs (279), it is likely that other metabolites elevated during or after exercise have important roles to play in tissue adaptation. Understanding how circulating metabolites trigger structural and functional changes in the heart could lead to the development of novel therapies to improve cardiac health.

## Summary

Metabolic changes caused by exercise are important for cardiac remodeling and adaptation. The integrative metabolic changes brought forth by exercise combine with changes in cardiac workload to regulate cardiac metabolism. In particular, exercise alters levels of competing substrates, and it changes the abundance of circulating hormones, which cue metabolic pathways that are critical for transcriptional changes and cardiac growth. In addition, changes in circulating and endogenous metabolites can trigger physiologic growth by activating prohypertrophic signaling pathways. Nevertheless, numerous questions remain, including questions of how to optimize the amount of exercise to produce beneficial, as opposed to deleterious, effects on cardiovascular health (280) as well as mechanistic questions of how exercise-induced changes in metabolism couple the synthesis of structural materials to activation of the physiological cardiac growth program (118). While this knowledge is acquired, it appears that we would be best served by sticking to the advice of the ancient Greeks—“Exercise till the mind feels delight in reposing from the fatigue.”—Socrates.

## Author contributions

All authors listed have made substantial, direct, and intellectual contribution to the work and approved it for publication.

### Conflict of interest statement

The authors declare that the research was conducted in the absence of any commercial or financial relationships that could be construed as a potential conflict of interest.
